# Sustainability of Long-term Care: Puzzling Tasks Ahead for Policy-Makers

**DOI:** 10.15171/ijhpm.2016.109

**Published:** 2016-08-17

**Authors:** Ilaria Mosca, Philip J. van der Wees, Esther S. Mot, Joost J.G. Wammes, Patrick P.T. Jeurissen

**Affiliations:** ^1^Division of Health Systems and Public Health, World Health Organization Regional Office for Europe, Copenhagen, Denmark.; ^2^Ecorys Netherlands B.V., Rotterdam, The Netherlands.; ^3^Radboud University Medical Center, Nijmegen, The Netherlands.; ^4^Radboud Institute for Health Sciences, Nijmegen, The Netherlands.; ^5^Celsus Academy for Sustainable Healthcare, and Scientific Institute for Quality of Healthcare, Nijmegen, The Netherlands.; ^6^CPB Netherlands Bureau for Economic Policy Analysis, The Hague, The Netherlands.

**Keywords:** Long-term Care (LTC), Healthcare Reform, Belgium, England, France, Italy, The Netherlands

## Abstract

**Background:** The sustainability of long-term care (LTC) is a prominent policy priority in many Western countries. LTC is one of the most pressing fiscal issues for the growing population of elderly people in the European Union (EU) Member States. Country recommendations regarding LTC are prominent under the EU’s European Semester.

**Methods:** This paper examines challenges related to the financial- and organizational sustainability of LTC systems in the EU. We combined a targeted literature review and a descriptive selected country analysis of: (1) public- and private funding; (2) informal care and externalities; and (3) the possible role of technology in increasing productivity. Countries were selected via purposive sampling to establish a cohort of country cases covering the spectrum of differences in LTC systems: public spending, private funding, informal care use, informal care support, and cash benefits.

**Results:** The aging of the population, the increasing gap between availability of informal care and demand for LTC, substantial market failures of private funding for LTC, and fiscal imbalances in some countries, have led to structural reforms and enduring pressures for LTC policy-makers across the EU. Our exploration of national policies illustrates different solutions that attempt to promote fairness while stimulating efficient delivery of services. Important steps must be taken to address the sustainability of LTC. First, countries should look deeper into the possibilities of complementing public- and private funding, as well as at addressing market failures of private funding. Second, informal care externalities with spill-over into neighboring policy areas, the labor force, and formal LTC workers, should be properly addressed. Thirdly, innovations in LTC services should be stimulated to increase productivity through technology and process innovations, and to reduce costs.

**Conclusion:** The analysis shows why it is difficult for EU Member State governments to meet all their goals for sustainable LTC, given the demographic- and fiscal circumstances, and the complexities of LTC systems. It also shows the usefulness to learn from policy design and implementation of LTC policy in other countries, within and outside the EU. Researchers can contribute by studying conditions, under which the strategies explored might deliver solutions for policy-makers.

## Background

The sustainability of long-term care (LTC) is currently a prominent policy priority in many European Union (EU) countries. LTC for the growing population of elderly people is one of the most pressing fiscal issues in EU Member States. The European Commission estimates that public LTC expenditures will increase from 1.6% of gross domestic product (GDP) in 2013 to 2.7% by 2060.^1^ Rising expectations of the ‘baby boom generation’ around service provision add to the fiscal pressure. The European Semester is the EU’s annual cycle of economic policy guidance and surveillance, with country recommendations. Following the 2014 Semester, country-specific recommendations regarding LTC have become the focus of EU policy.^2^

Policies addressing financial sustainability in health generally depend either on increasing private funding (eg, limiting entitlements or increasing out-of-pocket payments) or increasing productivity growth and reducing unit costs. These options come with puzzling policy problems. First, many LTC systems depend substantially on informal care.^3^ Private payments also cover substantial amounts of care for the elderly. Second, increasing productivity might be difficult due to LTC’s dependence on high labor inputs, the so-called Baumol Effect.^4^

Private funding in LTC implies out-of-pocket payments and other private funding arrangements. However, such arrangements may have serious, unintended consequences and are often inaccessible to people with lower incomes. In effect, it might mean that consumers become more dependent on informal caregivers. A growing informal care gap will then emerge, as the demand for informal care is expected to increase faster than its supply.^3^ Externalities of informal care on caregivers’ health and employment – in the form of opportunity costs or forgone wages – can be quite significant. Labor immigration from new EU Member States and developing countries could fill this gap, but can lead to problems, such as a gray labor market, which is visible in countries with fewer formal LTC entitlements.^3^

Policy-makers, who wish to avoid such options, are dependent upon policies that substantially increase productivity growth and reduce unit costs. However, most LTC services are labor-intensive and significant productivity improvements are hard to achieve. In some countries, policies focus on increasing competition and marketization in LTC.^5^ LTC might also benefit from a greater use of technology or process innovations to achieve productivity gains. A much sought-after solution is to increase efficiency by reducing expensive institutional stays and substituting these with low-cost home care.^3^

This paper examines the challenges of LTC systems in the EU, with the aim of identifying potential mechanisms for policy-makers to enhance sustainability. We combined a targeted review of the literature with a descriptive analysis of selected EU countries. The analysis describes public- and private funding, informal care, and possible productivity gains from new technology and process innovations in LTC. For each of these issues, we present country-specific cases and policies to show and compare different approaches towards addressing the long-term sustainability of LTC. We also explore avenues for future policy. This can contribute to the development of a grounded framework for analyzing sustainability in LTC.

## Methods

### Literature Review

We reviewed the literature to: (*a*) find studies that compared LTC systems; (*b*) identify policy measures for LTC sustainability, and (*c*) study the impact of policy measures on LTC sustainability. We performed a targeted literature search in PubMed and Google Scholar to find both peer-reviewed publications and gray literature (eg, reports by governments, independent organizations). We used combinations of the following search terms: long-term care, long-term-care financing, informal care, formal care, workforce, public funding, private funding, financial sustainability, organizational sustainability, aging, substitution, productivity, marketization, and competition. In addition, we hand-searched identified publications for relevant references and websites of policy institutions.

The literature review led us to formulate three important policy challenges for EU countries: (1) how should public and private funding of LTC be combined to guarantee fiscal sustainability in the long run; (2) how should the increasing gap in the availability of informal care and the externalities (ie, ‘spill-over effects’) on caregivers be addressed; and (3) how can LTC productivity be improved. We, therefore, focused on descriptive selected country analysis related to: (1) public- and private funding; (2) informal care and externalities; and (3) the possible role of technology and process innovation in increasing productivity.

### Country Selection

LTC systems differ greatly between EU countries. We selected Belgium, England, France, Italy, and the Netherlands, to cover most of the differences between healthcare systems in the EU. There is a lot of variety in public spending, private funding, informal care use, informal care support, and cash benefits.^6^ This hinders sampling according to the principle of health systems that are most similar in terms of certain critical variables. Using broadly collected indicators of LTC requirements — such as the percentage of the population over the age of 80, the percentage of adults over the age of 65 with strong limitations in daily activities, or the prevalence of dementia ­— only increases the large inter-country diversity.^7^ Public expenditures for LTC also vary greatly between countries. Such evidence adds up to the fact that LTC systems are very much embedded in national systems that have followed their own path dependency.

We searched for countries that differ in important aspects of their health systems to provide a variety of case examples. England is widely recognized as the archetypal National Health Service (NHS) system, while Italy has a decentralized NHS, as well as a more corporatist culture. France has a single insurer Bismarck system, while Belgium relies on multiple insurers. Over the past decade, the Netherlands has evolved from a Bismarck country, toward a more market-based model.

## Results

Table 1 provides detailed insights on public- and private funding and coverage for LTC.

**Table 1:** Financing and Coverage

	**Belgium**	**England**	**France**	**Italy**	**The Netherlands**
Public expenditure on LTC as a percentage of GDP (2013)	2.1%	1.2%	2.0%	1.8%	4.1%
Eligibility for coverage	Universal coverage within a single system (health-related and personal care). Social care (domestic care and other support) is a regional responsibility	Mixed: means-tested social care system with universal benefits for disability	Mixed system	Mixed system	Universal coverage within a single system (until 2015)
Coverage programs	Via the health system, a federal program, Flemish and regional programs	Means-tested, safety net (adult social care) and universal benefits (DLA and AL)	Income-related benefits (Said, APA)	Parallel universal scheme (institutional care [RSA] as part of the health system and care allowances [IA])	Public LTC insurance model – social insurance (Long-Term Care Act)
Private LTC insurance availability and type	Complementary mutual health insurance (reimbursement policies)	Life annuities are offered. Less than 0.05% of people aged 40+ hold a private insurance policy	Mainly indemnity policies: 15% of people aged 40+ hold a private insurance policy	Indemnity policies	No
Policies to increase the level of private insurance	No	No	Group insurance policies are offered. LTC insurance policies are offered as part of life insurance policies	No	No

Abbreviations: LTC‏, long-term care; GDP, gross domestic product; DLA‏, disability living allowance; AL, attendance allowance; RSA, Residenze Sanitarie Assistenziali; IA, indennità di accompagnamento. Source: OECD Health Data^8^; Colombo et al^3^; ENEPRI Research Report No. 117, based on SHARE wave II, and different country reports.^9^

Public expenditure on LTC, as a percentage of GDP in 2010, ranged from 1.2% to 4.1% in these countries. Funding systems also varied considerably. The weighted percentage of elderly people using personal care by informal caregivers ranged from 3.2% to 14.6% in our country sample.^3^

### Public- and Private Funding

A recurring question for many countries is: How to fund LTC in the long run? A key aspect of this challenge is the balance between public- and private funding. Aging of the populations and the increasing financial pressures on national budgets are spurring countries to look for alternatives to fund LTC. Thus, there is a growing interest in the potential role of private funding.

There are essentially two ways to increase private funding. The first way is to increase the direct financial contributions of consumers (eg, via means testing, savings, or reverse mortgages). Since pensions are generally lower than wages, and there is a highly skewed actual distribution of wealth, substantial numbers of care seekers will not be able to fall back on such sources. The second way is to create a market for private LTC insurance. We illustrate the problems of means testing and private insurance coverage by exploring the current experiences of England and France.

### Means Testing

In England, universal coverage aims to protect people, who incur very high LTC costs. Towards this aim, England relies on means testing for public funding of LTC. Patients from lower income households are granted higher subsidies. Only individuals with income and assets below the means-tested level receive publicly funded social care. The system also directs services toward those who live alone and who do not receive informal care.^10^ Individuals’ LTC needs are assessed by the social service departments of local authorities. Under the current system (2013), people with assets (savings and capital) above £23 250 are not eligible for local authority support and must pay their costs privately. Individuals with assets below the threshold are financially supported by the State, although they are required to pay a share of the costs of their care.^11^ The means-testing policy suggests that elderly citizens with moderate means face the risk of extremely high LTC costs.

In 2011, the Dilnot Commission proposed increasing the threshold for residential care and capping the lifetime care costs people face.^12^ Based on these recommendations, the English Government set the upper threshold for means testing in residential care to £118 000. Moreover, a cap of £72 000 on the total amount of costs for eligible care and support needs was recommended.^13^ The higher cap increases the affordability of LTC for middle-income people, but still fails to protect a substantial share of elderly people against significant costs. According to the plan, the new system was to become effective in April 2016.^14^ However, the necessary allocated funds were reshuffled to fight the acute funding gaps in social care, and the plan has been deferred to 2020. In conclusion, by aiming to offer comprehensive services to people with lower incomes, the English system fails to prevent high fiscal burdens for the middle class, due to extremely high private costs.

### Private Insurance

While private funding has attracted considerable interest in recent years, the private LTC insurance market has not yet gained a large role. Recent figures show that — except in the United States^15^ and France^16^ — private insurance accounts for less than 2% of total LTC spending.^3^ With public funding coming under pressure, there is an interest in better understanding how private insurance mechanisms could complement public coverage. Below, we discuss several reasons (ie, market failures) for the low uptake of LTC insurance.

On the demand side, limited consumer rationality or individual ‘myopia’ may play a role. Research shows that individuals find it difficult to understand low-probability high-loss events such as LTC. Moreover, the existence of public LTC insurance, the availability of public support, and the possibility of informal care can distort an individual’s willingness to obtain private insurance.

It can also be considered rational to not buy LTC insurance. Pauly^17^ explained this rationality by noting that LTC insurance would substitute formal care for preferred informal care by neighbors, family members, or the community. This argument however, was objected to by Cramer and Jensen,^18^ who found that income stood out in predicting purchase of LTC insurance in the United States. In France, purchase is partly motivated by a desire to protect family members from having to provide care.^19^

Adverse selection and moral hazard certainly play a role, as insurers may limit eligibility for private LTC insurance to individuals without pre-existing conditions, or set high, risk-adjusted prices. Another barrier to purchasing private LTC insurance is the competing financial obligations that individuals and families may face. Low-income households simply cannot afford private coverage. Brown and Finkelstein^20^ empirically showed that in the United States, LTC policy premiums are substantially higher than the expected benefits, which reduces monetary value for policy holders. On the supply side, market functions may be impaired by the insurers’ limited ability to control the covered LTC risk, because of future uncertainty and limited competition. This, in turn, can lead to premium volatility. To guarantee the insurers’ financial viability, premiums are subject to higher prices, if the risk pool of insured increases.

In France, the popularity of private LTC insurance steadily grew in the early 2000s, until in 2012 it covered 11% of the adult French population.^16^ French private LTC insurers offer a variety of products that may be purchased individually or collectively. Policies tend to be cheap, but also to offer low benefits. Individual policies cost €345 on average in 2010, whereas average monthly payouts equaled €540. Employer-based polices cost €74 annually on average, and monthly payouts equaled €150. Due to these limited benefits, in case of dependency, people with LTC still depend on ‘safety-net programs’ or must pay the majority of costs privately. French people with pre-existing conditions are often exempted from insurance.^16,21^ In 2001, the growth rate in the number of insurance contracts in France reached 22%, mainly because insurers started marketing or cross-selling their products. The growth rate declined in 2006, after the French Government announced its intention to reform LTC insurance.^22^

In addition, almost 75% of French LTC insurance contracts were purchased under a group insurance plan.^16^ Group insurance offers several advantages, including the mitigation of adverse selection. Risks are spread over a large group, which allows for better accessibility, fewer exclusions, and greater negotiation power for insurers vis-à-vis providers. On the other hand, since most group contracts are employer-based, they also limit the availability of LTC insurance for people who are not currently working. The French Government attempts to increase individual financial flexibility by combining LTC insurance with other financial products.^23^ The program *vente en viager*, for example, combines the sale of an elderly person’s home with their right to live in it (or receive rent) until their death.^24^ Such reverse mortgage products, however, are highly sensitive to fluctuations in housing prices. They work well when house prices increase, but can have dramatic consequences when they fall.^25^

Nevertheless, the French experience shows that it is possible to build a significant, private market. However, the case also illustrates that LTC insurance forms only a partial solution for a minority of the population and requires complementation.

### Informal Care and Externalities

Informal care forms the backbone of LTC and is, thus, the natural alternative when formal arrangements come under fiscal pressure. Informal care provides several benefits. First, the availability of informal care can allow people to remain (or, at least, remain longer) in their homes. This might have positive consequences for individual well-being and can be emotionally beneficial. Second, by staying at home longer, LTC costs might be lower than if the person were institutionalized. There is, therefore, a trend to invest public resources into personal budgets or cash benefits for LTC and/or to allow some tax benefits for informal caregivers or those receiving LTC.^3^ Apart from stimulating informal care, cash benefits may serve a wide number of policy goals, such as income support for carers, to provide recipients more control on the care they receive (in choosing the service they need, from their preferred providers, according to their own conditions), to stimulate (an underdeveloped) formal workforce, or to increase allocative efficiency of the system. Below, we primarily discuss cash benefits as a means of stimulating informal care, since our article focuses on fiscal sustainability, and monetizing informal care adds to the public purse, and we report on other potential goals, if applicable.

Table 2 shows that each of the selected countries adopted some policy measures for supporting informal caregivers. However, scale and scope are limited. This is supported by Triantafillou et al,^26^ who concluded that the basket of relevant services to empower caregivers by enhancing skills and knowledge is not highly developed. Moreover, knowledge about informal care support is very limited, especially concerning effectiveness on mitigating adverse effects of caring on work and health (mental and physical).^3^ Courtin et al^27^ also reported that EU policies are at an early stage of development. They showed that various policies have been implemented in EU countries: financial support is the most common, followed by respite care and training. Moreover, most EU countries do not systematically identify informal caregivers, or assess their needs.^27^

**Table 2:** Informal Care Support

	**Belgium**	**England**	**France**	**Italy**	**The Netherlands**
Allowance to caregiver	*Care premium (mantelzorgpremie).* Eligibility criteria and payment conditions vary	Caregiver’s allowance for those spending at least 35 hours per week providing care	No	No	No
Allowance to recipient	Integration allowance; income and needs tested	Al, for those who need care for more than six months	Personal allowance (*Allocation Personnalisée à l’autonomie*) for people aged 60+, depending on disability and income	AL, needs tested	Personal budget, no age limit or income test to claim
Tax support	No	No	Planned tax reductions for hiring formal labor	No	No
Paid leave	Palliative care leave up to two months and medical assistance leave up to 12 months. Time credit one to five years	No	Family solidarity leave for three months. For first-degree relatives or terminally ill co-residential member	Unknown	Leave for care up to 10 days, employers can refuse on serious business grounds. Paid 70% of earnings
Unpaid leave	Emergency leave: 10 days in private sector and 45 days in public sector	Emergency leave to care for a family member. The length should be reasonable (ie, 2 days)	Family support leave for three months	Unknown	50% of the number of hours worked, for 12 weeks in one or several periods
Policies to stimulate caregivers’ physical- and mental well-being	Training/education, respite care, and counseling	Training/education, respite care, and counseling	Training/education, respite care, and counseling	Unknown	Training/education, respite care, and counseling (such as the POM method: preventive counseling and support)

Abbreviations: POM, reventieve Ondersteuning Matelzorgers; AL, attendance allowance. Source: OECD Health Data^8^; Colombo et al^3^; ENEPRI Research Report No. 117, based on SHARE wave II, and different country reports.^9^

The ratio of available informal care workers — predominantly female family members — to care seekers is diminishing in many countries.^3^ This is caused by a decrease in the share of the working-age population and a number of societal changes (eg, declining family size, decreased co-residence of elderly people with their children, higher divorce rates, greater female labor market participation, increased retirement age, and a possible decline in the willingness to care).^3^ This is especially the case for people with higher incomes, as they have larger opportunity costs.

There are also disadvantages for the caregivers. Career interruptions become frequent when one has to look after someone else, which leads to a deterioration of human capital or simply fewer opportunities for a better career.^3^ In addition to such financial losses, a large body of research has found that care giving has negative effects on health. Psychological distress, isolation, and lack of support, are drivers in the worsening mental health of informal caregivers, particularly when intensive and co-residential care is provided.^28,29^ There is less evidence available on the detrimental effects of providing less intensive care on mental health. These negative effects on the caregiver also seem to differ across countries.^3,30^

Formal- and informal care can be considered substitutes or complements, depending upon the type of care and the person’s needs. Care responsibilities that depend on a higher level of education generally require more formal workers. Bolin et al^31^ consider informal care to be a substitute for formal care in domestic help, and complementary to nursing care. There is also evidence that suggests that personal care may be substituted for informal care.^32,33^ The policy in many countries is aimed at shifting institutional care towards a combination of formal- and informal care for people living at home.

For instance, over the last decade, Belgium has implemented several arrangements to stimulate older people to live independently at home for as long as possible, while guaranteeing access to affordable formal care services. Arrangements are aimed at shifting residential LTC from low-care to high-care dependents, and include the establishment of alternative types of care to support home care (including nursing care). These reforms have resulted in a relative shift toward living at home, rather than in nursing homes, with a 30% increase in home care and only a limited increase in nursing care (11%). In addition, the supply of semi-residential services, such as day-care centers and short-stay centers, has increased.^34-36^ However, despite these measures to reduce residential care and stimulate home care, the number of residential care users is projected to further increase due to the aging of the Belgian population.^35^

Several countries rely on multiple measures to support the work of informal caregivers, to mitigate the adverse effects of informal care on work and health, and to acknowledge their important contribution to overall LTC. Cash benefits are either financial measures paid directly to the carer to help sustain their ability to provide care, or cash benefits are given to the care recipient, wherein recipients are stimulated to choose the care they need and/or to give control on the care they receive. Other supporting arrangements that may support informal workers – but not always exclusively aimed at the informal worker – include respite care; tax benefits; home-based, professional, formal services; home support devices; home adaptations; paid- or unpaid leave; counseling and training services; and information and coordination services.

It is quite complicated to set the amount of a care allowance. Usually, the income level for care allowances is fairly low, as ‘market level’ amounts would compete with other forms of employment.^26^ In particular, caregivers with little education, who experience difficulties in entering the labor market, could be discouraged to look for a job outside the house. From this perspective, care allowances might appear to be instruments that grant some form of income assistance, while maintaining care giving as a low-profile and low-paid job.^3^

In England, pilot studies have found tension between competition, market stability, choice, and (female) caregivers’ roles. For example, systems with health insurance brokers resulted in increased costs and care delays, and local authorities placed only a few home care providers on their framework agreement. In addition, many elderly people may not want to act as empowered consumers of care.^37^ However, an earlier study found that individual budgets can have positive effects on caregivers (who are predominantly female). These caregivers reported an increased quality of life, were more likely to be fully occupied in activities of their choice, and were more likely to have social lives. Individual budgets, thus, provided choice, and, as such, recognized caregivers’ rights and support needs.^38^ Other literature shows that LTC coverage expansions go ‘hand-in-hand’ with increased cost sharing by families and subsidization of informal care by families. As such, the implicit partnership of cost sharing and incentivizing family care giving may serve as a catalyst for coverage expansion.^39^

The administrative burden of supporting informal care may be considerable, but fiscal stress necessitates administrative control and monitoring. In terms of administrative tasks, it is difficult to monitor several requirements, such as identification of an eligible caregiver, ensuring the necessary care effort, and maintaining the relationship. Moreover, in the absence of regulation, care allowances may encourage black markets.^40^ Fiscal sustainability is at stake in many countries, including France and the Netherlands. Germany is one of the countries that has been most successful in maintaining fiscal sustainability by using a reliable and strict assessment process. Its flexible policy also allows officials to make minor adjustments (such as increasing contribution rates).^40^

In contrast, attendance allowances (ALs) have several advantages for policy-makers. First, cash benefits can be used to increase the autonomy of care recipients by allowing flexibility in organizing the type of care people want. Second, the eligibility requirements are more clear-cut than care allowances. Cash benefits or vouchers are provided to individuals, who have undergone an assessment. Third, cash benefits can be used to control costs, as the value of the benefit is usually set below the cost of in-kind services. However, the effects on cost control are heavily dependent upon the eligibility criteria for cash benefits and the way that the system is designed. In the Netherlands, the introduction of potentially very high cash benefits for a broad group of older- and handicapped individuals has increased the total cost of LTC, whereas England, France, and Italy, did not have these problems with their systems of cash benefits. Some studies from the Netherlands show that the allowances tend to appeal to a group of people who would have not applied for care (in-kind) if they had not had the option of having their own budget.^41^

In 1995, the Netherlands started to offer personal budgets for people entitled to LTC. Compared to many other countries, the eligibility criteria were quite generous. For example, they included people who needed only home help, because of their limitations, or a few hours of help every week towards organizing their lives. The amount of the personal budget was aligned with the costs of in-kind care. Initially, the reimbursed level was set at 70% of the formal rate of LTC. Thus, recipients could be eligible for benefits that equaled tens of thousands of Euros each year, or even more in severe cases. Patients value the personal budgets as an effective means to purchase and organize care. A tenfold growth in the number of budget holders led to an average 23% yearly cost increase (up to €2.2 billion in 2010).^42^

A considerable part of the personal budgets turned out to be used for complementary services and not so much for substitution of care in-kind. Increased uptake has largely been for the benefit of children and adolescents with learning disabilities, autistic spectrum diagnoses and intellectual disabilities, who previously received informal (unpaid) care.^42^ To counteract the problem of an exploding demand for personal budgets, stricter needs assessment protocols were implemented.^43^ In 2015, a major reform of the Dutch LTC system took place.^44^ The Dutch Government chose to maintain the option of cash benefits for all categories of care recipients, but stricter criteria were introduced and cash benefits are no longer transferred to the bank account of the budget holders; instead they received the right to purchase care that is paid by a third party. Reimbursement levels – relative to in-kind services - may vary, but are required to be sufficient for purchasing care.^45^

In stark contrast to the Netherlands, the LTC system in England is a ‘safety-net’ type of system, in which publicly funded care is only available for those with serious care needs and low incomes who cannot afford to pay for the care themselves.^10^ This restricts the group of individuals potentially entitled to cash benefits, such as direct payments and individual budgets. Both direct payments and personal budgets were primarily aimed at care recipients purchasing their own care. In 2007, the number of personal budget holders was about three times larger in the Netherlands than in England, despite the much smaller population of the Netherlands.^41^ Holders of direct payments appeared to have control and choice in organizing their care compared to personal budget holders.^37^

The French ‘cash-for-care’ scheme — *‘Allocation personnalisée d’autonomie’* (APA) — is strictly regulated.^46^ The cash benefits are aimed at people 60 years of age and older with a medium- to high level of dependency. The maximum allowance for the highest level of dependency was €1261 per month in 2012. The benefit must be spent according to a care plan developed by professionals employed by the French local authority. Due to income-dependent co-payments, APA recipients with a higher income fund a large part of the care plan themselves.

In Italy, cash benefits or companion payments (‘*Indennità di accompagnamento’*) are given to individuals. However, these individuals must be 100% disabled, must not be self-sufficient, and must not reside in an institution. All users receive the same amount of benefits, and each user may spend €487 per month on whatever he or she wants. Many recipients use the companion payment to pay migrant care workers who provide care.^47^ Vouchers are also used to buy healthcare services, either from an accredited provider (in some regions in Italy) or directly. Personal budgets also fall into this category. Care recipients can use the cash to pay an informal caregiver. The use of the cash benefit scheme increased by 75% in the last decade, while the number of home care users rose by 23%, and the number of residential care users remained stable. Currently, public expenditure on cash benefits (0.56% GDP) equals the sum of both expenditure on in-kind home services (0.25% GDP) and residential care (0.31% GDP).^3,47^

The cases of England, France, Italy, and the Netherlands have important differences with potential impacts for further policy. The cash benefits in England, France, and Italy aim to provide extra financial support in LTC systems characterized by low public spending and intensive informal care. As such, the benefits protect those in greatest need from catastrophic costs. On the contrary, the main purpose of personal budgets in the Netherlands was to improve the autonomy of patients — including those with less severe needs — in an already very accessible system, with high public spending and a large role of formal care. The personal budget system was also aimed at increasing providers’ responsiveness to patients’ needs (as was the case in England and elsewhere). Policy-makers realized at the time of implementation that the cash benefits might be used to pay informal caregivers, who already supported the budget holders. The Dutch approach aimed at increasing the quality of care in a highly accessible system, even for those with lower needs.

In conclusion, from a financial sustainability point of view, while the English, Italian, and French systems seem to work for creating additional capacity in a stretched LTC system at comparatively low costs, the Dutch personal budgets simply seem to pay for informal care that was already given without payment, or to add complementary services on top of a broad level of entitlements for a considerable amount of additional spending. Finally, the relatively low uptake and development of non-allowance policies, such as respite care and training, suggests ample opportunity for improvement. Nevertheless, opportunity costs for informal care providers are important in considering further policy stimulating informal care.^48^

### Productivity Gains

LTC is sensitive to the Baumol Effect, with wages rising in line with the general economy, even though LTC does not achieve significant productivity gains. This results in an increase in costs for a given level of output.^4^ The combination of population aging, lower productivity gains than the rest of the economy, and the greater labor intensity of LTC, raises the question of how to handle the long-term financial sustainability of LTC. One possibility would be to benefit from a greater use of technology or process innovations.

Evidence across the Organisation for Economic Co-operation and Development (OECD) countries shows that there is more potential for using technology (especially information technology) in LTC. Mori et al^49^ distinguished between two general classes of available technological solutions: devices and aids, and ICT. Such solutions enable increased patient engagement and self-management by promoting citizens’ abilities to effectively manage their own health. Relations between the various actors (care recipient, formal and informal caregiver) are also altered by the use of technology. However, most studies that assess the use of technology in LTC are still based on pilot programs; further assessment of these programs is needed to understand the main findings and possibly enlarge their scope of application.^3^

This point is confirmed by Mazzeo et al,^50^ who looked at initiatives to support development and diffusion of technology to improve LTC quality. In Italy, for example, the Telemedicine/e-Health services comprise of 80 projects spread throughout the country. There is, however, no national strategic plan for telemedicine or e-Health. There are also no payment mechanisms that stimulate the use of telemedicine services. In the Netherlands, Telecare services that consist of a 24/7 television contact between the patient and the medical center, started as a regional program.

To facilitate the diffusion of technology, there is a need to address infrastructural readiness, investment costs,^51^ and resistance to change by LTC workers.^52^ Mori et al^49^ identified barriers to implementing technological innovation in LTC: organizational inertia, barriers related to privacy and security, the lack of coherence with the regulatory system, and the issue of data management accountability. However, technological help has often been perceived in LTC as a complement to the labor workforce, rather than a substitute.^53^ A recent multicenter study in England found that the gains of people using telehealth in addition to standard support and treatment were small or similar to those receiving usual care, and that the total costs for the telehealth group were higher than for the usual care group.^54-56^

To date, there is still little evidence about persistent productivity improvements in LTC. Dumaij^57^ offered an interesting overview of productivity developments in Dutch nursing homes, residential homes, and home care, between 1972 and 2010, that focused on the effects of policy changes on LTC productivity. This empirical analysis found that the change in funding system in the LTC sector — from a fixed fee per bed/patient to performance-oriented funding — did little to increase overall productivity. This could partly be a consequence of increasing case-mix levels that are not adequately adjusted for.^57^

In conclusion, effective technical tools to improve productivity in LTC do not yet appear to be available. It is difficult to substitute labor for capital. Most of the innovations developed in the past years to stimulate productivity were targeted at nursing- and home care. These included home care technology; tools for mobility; social monitoring; the development of many new types of small-scale residential care homes; collaborations between volunteers and informal care; self-steering teams; and smart planning and work processes.^57^ According to Mori et al,^49^ the phenomenon of technology-assisted LTC is still in its infancy, mostly because industry involvement is still largely underdeveloped, due to the inadequacy of the highly fragmented demand side.

## Discussion

The aging of the population, the increasing shortage of informal care, the substantial market failures for private funding of LTC, as well as fiscal imbalances in some countries, have led to enduring pressures for LTC policy-makers across the EU. Greater productivity through technology or process innovation might be the most attractive solution, since decreasing unit costs enable the avoidance of difficult political decisions about cutting the level of publicly funded services. While increasing productivity with the aid of technical facilities or incentives (market-based or otherwise) may be politically attractive, the actual implementation of such strategies is difficult, and may sometimes, in fact, increase costs. Key challenges — community care, assisted living technologies, support of informal workers, and mobilizing volunteerism in nursing homes — can all be stimulated with cash benefits, but these come with complex design issues. When prefunding is needed, such as for ICT investments, central funding often remains necessary.

Although there are still possibilities for stimulating informal care (e.g. cash allowances, respite care, support services, leave from work), the cost-effectiveness of such measures is not always clear. It is extremely important to find ways to stimulate informal care. This is very much about finding affordable ways to build ‘affectionate’ communities, and in supporting families in helping each other. However, many countries already heavily rely on informal care workers, and it is hard to see how informal care can accommodate rising demand without some negative consequences. Taking people (predominantly women) out of the workforce not only reduces tax revenue from earned income, but also increases their long-term risk for financial dependence on the government.

The boundaries of LTC and other sectors, such as social care, housing, social security (pensions), and acute healthcare, are often somewhat blurred. Elderly people are entitled to multiple public services, which might offer opportunities to shift costs to neighboring policy areas. It also makes LTC systems vulnerable to a never-ending number of policy reforms intended to increase the efficiencies of scope between the different sectors. For example, the Netherlands witnessed a new reform (shifting parts of LTC to the more competitive arrangements of acute care and other parts to social care in the municipalities) that left a much smaller inpatient sector as the core of public insurance for LTC.^30,44,58^

An increase in private funding (including self-funding) is potentially a substantial source for future LTC spending. However, this option is not per se feasible. Solutions differ by country and require further development. Due to the market failures of private insurance and savings policies, it would be appropriate for a government to step in and arrange, for example, tax credits to build a minimum scale. Tax credits might persuade those who can afford such products to contribute some personal resources. However, this only contributes to financial sustainability if these tax benefits are ‘repaid’ through either fewer publicly funded benefits or, as an alternative, higher means testing (such policies will probably become disproportionally bought by higher income people). Currently, many LTC systems substantially rely on private self-funding. Means testing is often used to cover for a substantial share of the necessary public resources. However, this is often an unattractive deal for wealthier people, who share a large chunk of the financial burden for comparatively moderate service levels.

The trade-off between comprehensive services for the most needy and a limited service provision for the middle-class seems to be one of the major issues when LTC systems come under increasing fiscal pressures. The political unpopularity of such strategies can be illustrated with the case in England, where means testing now comes with proposals for a threshold on cost-sharing. Policy-makers in countries with underfunded LTC services could contemplate a system of limited cash benefits to stimulate patient-specific choices and fill gaps, as in Italy.

## Conclusion

Our exploration shows that for LTC to be sustainable in the long run, important steps must be taken. First, private LTC insurance forms only a partial solution for a minority of the population, and must be complemented with public programs. EU countries should look into the possibilities of complementing public- and private funding, and address the market failures of private funding. Second, informal care is essential in elderly care, but it needs to anticipate societal changes, such as greater female labor market participation. Policies should be aimed at maximizing the informal care reservoir, while addressing potential negative consequences in order to avoid even greater fiscal imbalances. Informal care’s externalities into the broader labor force and LTC’s externalities into neighboring policy areas, such as social care, housing, and social security (pensions) should be considered and properly addressed. Third, increasing LTC productivity through technology or process innovation is a major challenge. Innovations to increase the efficiency of LTC services should be stimulated, especially if they coincide with lower costs. A promising approach is to consolidate the fragmented demand side.

Our exploration of national policies illustrates a differing landscape of solutions that circle around fairness and stimulating efficient service delivery. Our analysis found that the complexity of the challenges offers no easy answers. The analysis shows the difficulty for EU Member State governments to meet all their goals for sustainable LTC, given the demographic and fiscal circumstances and the complexities of LTC systems. It also shows the usefulness to learn from policy design and implementation of LTC policy in other countries within and outside the EU. Without in-depth theoretical- and practical guidance, years of potentially unfruitful policy experimentation might lay ahead. Researchers can contribute by studying conditions, under which the strategies explored might deliver solutions for policy-makers.

## Acknowledgements

This study was sponsored by the Dutch Ministry of Health, Welfare and Sports, The Hague, The Netherlands. The sponsor did not have a role in the study design, data collection and analysis, or writing of the report.

## Ethical issues

Our study did not involve human subjects (that included human material or human data), and required no approval from an ethics committee.

## Competing interests

PPTJ is employed by the Dutch Ministry of Health, Welfare and Sports, The Hague, The Netherlands. All authors declare that they have no other competing interests in conducting this study.

## Authors’ contributions

PJvdW and PPTJ were responsible for study concept and design. IM, PJvdW, and JJGW were responsible for data collection and drafting of the manuscript. JJGW, IM, PJvdW, ESM, and PPTJ contributed to analysis and interpretation of data. ESM and PPTJ provided important feedback to draft versions of the manuscript. All authors approved the final version of the manuscript.

## Authors’ affiliations

^1^Division of Health Systems and Public Health, World Health Organization Regional Office for Europe, Copenhagen, Denmark. ^2^Ecorys Netherlands B.V., Rotterdam, The Netherlands. ^3^Radboud University Medical Center, Nijmegen, The Netherlands. ^4^Radboud Institute for Health Sciences, Nijmegen, The Netherlands. ^5^Celsus Academy for Sustainable Healthcare, and Scientific Institute for Quality of Healthcare, Nijmegen, The Netherlands. ^6^CPB Netherlands Bureau for Economic Policy Analysis, The Hague, The Netherlands.

## Key messages

**Implications for policy makers**

- Private long-term care (LTC) insurance only offers a partial solution for a minority of the population, and European Union (EU) countries should consider complementing public- and private funding of LTC, and addressing the market failures of private funding.
- Informal care is an essential cornerstone of sustainable LTC, but it faces many challenges related to care allowances, attendance allowances (ALs), and opportunity costs for predominantly female informal care workers. If the supply of informal care cannot be sustained, formal elderly care systems will sustain greater fiscal imbalances.
- Increasing LTC productivity through technology is a major challenge. Innovations to increase the efficiency of LTC services should be stimulated, especially if they coincide with lower costs.

**Implications for public**

Long-term care (LTC) for elderly and chronically ill people is becoming more and more expensive due to the growing population of elderly people. In addition, a shortage of professional workers may become a problem. A solution that contributes to sustainable LTC is to more strongly support informal care by family or friends.
